# Fungal colonization with *Pneumocystis* correlates to increasing chloride channel accessory 1 (hCLCA1) suggesting a pathway for up-regulation of airway mucus responses, in infant lungs

**DOI:** 10.1016/j.rinim.2014.07.001

**Published:** 2014-07-30

**Authors:** Francisco J. Pérez, Carolina A. Ponce, Diego A. Rojas, Pablo A. Iturra, Rebeca I. Bustamante, Myriam Gallo, Karime Hananias, Sergio L. Vargas

**Affiliations:** a Programa de Microbiología y Micología, Instituto de Ciencias Biomédicas, Facultad de Medicina Universidad de Chile, Santiago 8380453, Chile; b Servicio Médico Legal, Santiago 8380454, Chile

**Keywords:** *Pneumocystis jirovecii*, Primary infection, Immunocompetent, Airway

## Abstract

Fungal colonization with *Pneumocystis* is associated with increased airway mucus in infants during their primary *Pneumocystis* infection, and to severity of COPD in adults. The pathogenic mechanisms are under investigation. Interestingly, increased levels of hCLCA1 – a member of the calcium-sensitive chloride conductance family of proteins that drives mucus hypersecretion – have been associated with increased mucus production in patients diagnosed with COPD and in immunocompetent rodents with *Pneumocystis* infection. *Pneumocystis* is highly prevalent in infants; therefore, the contribution of *Pneumocystis* to hCLCA1 expression was examined in autopsied infant lungs. Respiratory viruses that may potentially increase mucus, were also examined. hCLCA1 expression was measured using actin-normalized Western-blot, and the burden of *Pneumocystis* organisms was quantified by qPCR in 55 autopsied lungs from apparently healthy infants who died in the community. Respiratory viruses were diagnosed using RT-PCR for RSV, metapneumovirus, influenza, and parainfluenza viruses; and by PCR for adenovirus. hCLCA1 levels in virus positive samples were comparable to those in virus-negative samples. An association between *Pneumocystis* and increased hCLCA1 expression was documented (*P*=0.028). Additionally, increasing *Pneumocystis* burden correlated with increasing hCLCA1 protein expression levels (*P*=0.017). Results strengthen the evidence of *Pneumocystis*-associated up-regulation of mucus-related airway responses in infant lungs. Further characterization of this immunocompetent host-*Pneumocystis*-interaction, including assessment of potential clinical significance, is warranted.

## 1 Introduction

Mild and asymptomatic infections by the fungal pathogen *Pneumocystis* are of uncertain pathological significance. They are known as “*Pneumocystis* colonization” and are highly frequent in normal immunocompetent infants and adults [1]. Recent evidence of *Pneumocystis*-related pathology in immunocompetent infants with histologically mild and asymptomatic *Pneumocystis* infection, was provided by documenting increased protein levels of the goblet cell mucin MUC5AC, a marker of mucus, associated with *Pneumocystis* in autopsied lungs of infants who died in the community with an autopsy diagnosis compatible with Sudden Unexpected Infant Death (SUID) [2]. Mucus is widely recognized as an aggravating factor of respiratory illnesses, including chronic obstructive pulmonary disease (COPD), where *Pneumocystis* has been associated with increased disease severity [3]. Therefore, the documentation of *Pneumocystis*-related mucus pathology in infant lungs warrants continued research to elucidate whether *Pneumocystis* plays a role in the increased respiratory morbidity of infants characteristic of this age group [2].

Mucus production is stimulated through several intracellular pathways still under investigation; one proposed pathway is mediated by chloride channel accessory 1 (hCLCA1), a member of the calcium-sensitive chloride conductance (*CLCA*) family of genes, whose expression is increased in human airways of asthmatic and COPD patients [4–9]. In general, CLCA proteins mediate airway epithelium immune responses inducing mucous cell metaplasia and airway hyperreactivity [6,7]. More specifically, it has been documented in cell culture models, that *mClca3*/*hCLCA1* stimulates mucus (MUC5AC) production [7,10]. In addition, it has been shown in mouse models, and in human and rodent primary cell cultures, that *mClca3*/*hCLCA1* expression occurs through a Stat6-dependent pathway [8].

Important insight into the role of *Pneumocystis* in this pathway has been gained through studies using immunocompetent mouse models which showed that *mClCa3* (or *Gob5*), the murine homolog of *hCLCA1*, is significantly increased in association with *Pneumocystis*[11] . In addition, it has been documented more recently that *Pneumocystis* can induce STAT6-dependent pathways eliciting mouse-strain-dependent responses [12]. The link between CLCA proteins and mucus overproduction is well reported in animal models [6,7]. Studies in infant lungs would be ideal for understanding the link between *Pneumocystis* colonization and mucus overproduction recently reported in infants [2,13]. Moreover, since respiratory viruses are recognized agents of increased mucus production [4] and because their relative contribution to hCLCA1 and MUC5AC with respect to *Pneumocystis* in infant lung samples remains unknown, we also evaluated the presence of common respiratory viruses in infant lungs in this study.

## 2 Materials and methods

### 2.1 Subjects and samples

The study, approved by the Ethics Committees of the North Metropolitan Area of Health and of the University of Chile School of Medicine in Santiago, was retrospectively conducted in fresh-frozen stored infant lung specimens previously categorized as *Pneumocystis* negative or positive, blinded to autopsy diagnosis and date of death, by microscopy and n-PCR. Samples were age matched and a 1:2 (negative:positive) ratio was used. They corresponded to 55 legally-required infant autopsies conducted between 1999 and 2004 at the Servicio Medico Legal, the coroner’s office in Santiago. Samples stored at −80 °C, were selected from 18 *Pneumocystis*-negative and 37 *Pneumocystis*-positive infants with sufficient tissue left for analyses. Their mean age was 3.19 (1.0–11.9) months; all had died suddenly and unexpectedly (SUID) in the community without hospitalization [2]. One gram of deep lung tissue was extracted with all possible sterile precautions inside a laminar flow biosafety cabinet, flash-frozen pulverized in liquid nitrogen using a mortar and pestle, homogenized, and frozen at −80 °C until nPCR was repeated to re-confirm their *Pneumocystis jirovecii*-status. Quantitative PCR (qPCR) for *P. jirovecii* was performed on all *P. jirovecii*-positive samples; Reverse Transcription PCR (RT-PCR) or PCR for respiratory viruses, and Western blot analyses of hCLCA1 were also performed.

### 2.2 Pneumocystis and virus determinations

*Pneumocystis* status of samples was re-confirmed using a nested-PCR specific for *P. jirovecii* as described [2]. Total DNA extraction was performed using QIAamp^®^DNA Minikit (Qiagen, Valencia, CA, USA). RNA was extracted using Trizol reagent (Invitrogen, CA, USA) according to the manufacture’s instructions. *P. jirovecii* burden was quantified by qPCR amplifying the human *Pneumocystis* GpA/MSG gene with specific primers and probe (5′ d FAM-TGCAAACCAACCAAGTGTACGACAGG-BHQ-1 3′) as described [14,15]. These probe quantifications were compared with *Pneumocystis* SYBR green quantifications of the same specimens in our previous study [2]. cDNAs were synthesized to identify Respiratory Syncytial Virus (RSV), Influenza A and B, Parainfluenza virus 1, 2, and 3, and Metapneumovirus, by RT-PCR with specific primers [16–19]. Total DNA was used to evaluate Adenovirus by PCR as described [20]. Viral positive controls were additionally confirmed using standard diagnostic immunofluorescence microscopy. Bacterial cultures are not considered as part of the legal autopsy protocol, and were not done because the samples were received after 24 h post-mortem [2].

### 2.3 hCLCA1 determinations

Samples for hCLCA1 determinations were processed as described, unless stated otherwise. Western blot were performed from 30 µg protein aliquots, using SDS-PAGE 12% polyacrylamide resolving gels. hCLCA1 was detected using mouse anti-hCLCA1 IgG (1:500 sc-271156, Santa Cruz, USA). Measured values were normalized by human actin-gene expression for inter-sample comparison.

### 2.4 Statistical analyses

GraphPad Prism 5 software (San Diego, CA, USA) was used for analysis. Comparisons between normalized levels of hCLCA1 protein expression values according to the presence of *Pneumocystis* or of viruses were performed using Mann–Whitney. The correlation between hCLCA1 protein levels with *Pneumocystis* GpA/MSG copies was done using the Spearman test. A *P* value of <0.05 was considered significant.

## 3 Results

All selected infants were confirmed to have died suddenly and unexpectedly at home and without being hospitalized, indicating that *Pneumocystis* infection in them was mild. *P. jirovecii* diagnostic status was also re-confirmed by n-PCR in the 37 *Pneumocystis*-positive and 18 *Pneumocystis*-negative infants. Mean *Pneumocystis* burden, as determined using the probe method, was 10,119 (1–299,697; median 120) GpA/MSG copies/ng human DNA. *Pneumocystis* burden determinations using SYBR Green method, reported in a previous study on these same samples [2], were concordant with the probe method determinations in this study.

**Fig. 1:**
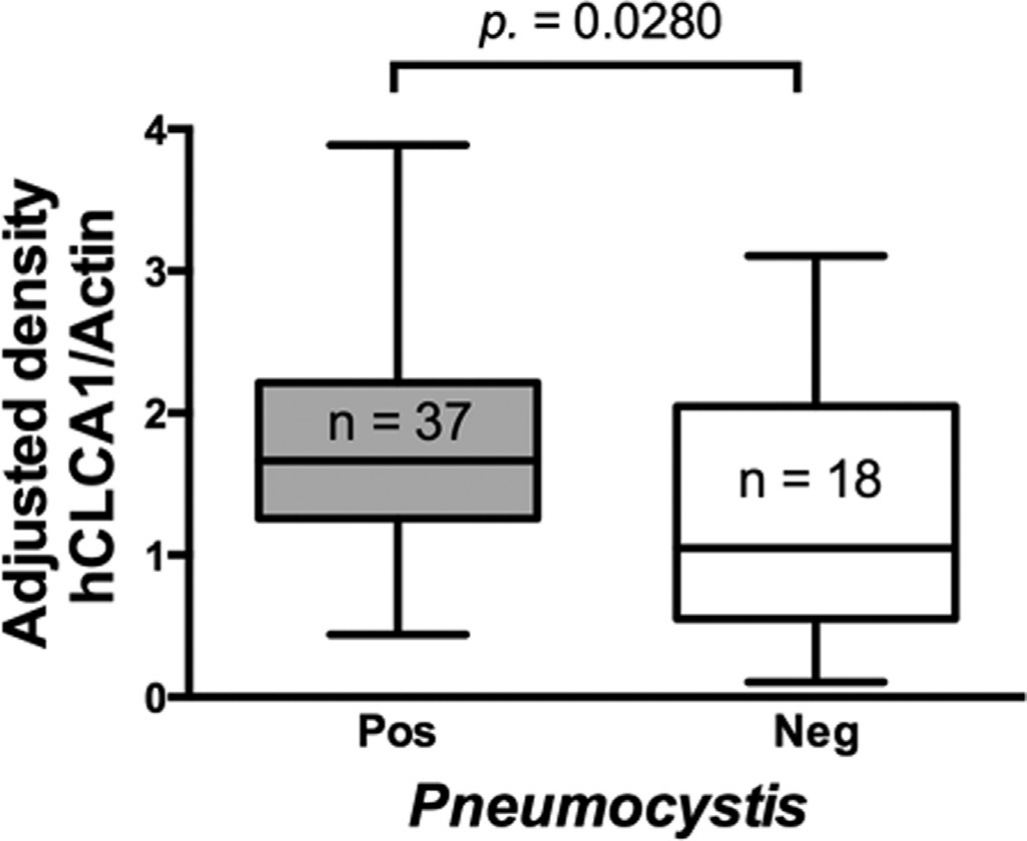
*Pneumocystis*-associated increased expression of hCLCA1 (*P*=0.0280). Western blot determinations performed in fresh-frozen homogenized autopsy lung samples from *Pneumocystis*-positive (*n*=37) and *Pneumocystis*-negative (*n*=18) infants*.* Data are presented as median interquartile range. hCLCA1 expression levels were analyzed using Mann–Whitney test. Significance was defined as *P*<0.05.

Analysis of protein extracts documented a significant increase in normalized expression levels of hCLCA1 in *Pneumocystis*-positive samples compared to *Pneumocystis*-negative samples (*P*=0.0280) (Fig. 1), suggesting that *Pneumocystis* is associated with airway epithelium stimulation including up-regulation of mucus-related responses.

**Fig. 2:**
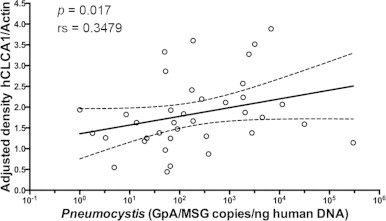
hCLCA1 expression positively correlates with *Pneumocystis* organism’s load (Spearman *r*s=0.34785; *P*=0.0171). Correlation graph of hCLCA1 protein expression levels compared with *Pneumocystis* burden as determined by qPCR. Linear regression was performed and the fitted line is showed on the graph. Broken lines represent 95% confidence intervals.

**Fig. 3:**
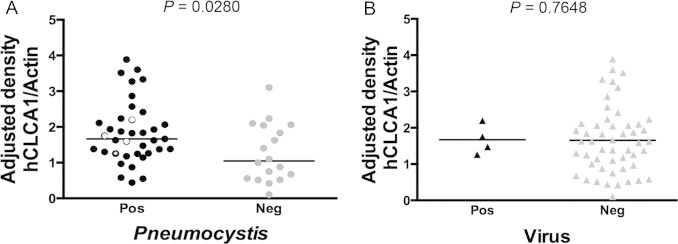
(A) Effect of viral co-infection on hCLCA1 protein expression in *Pneumocystis*-positive infant lung samples. Thin open circles represent RSV and thick open circles represent adenovirus. Horizontal black lines represent median values. (B) Expression of hCLCA1 protein in virus-positive samples (*n*=4) compared to virus-negative samples (*n*=51). Mann–Whitney test. Significance was defined as *p*<0.05.

The contribution of *Pneumocystis* burden to the expression of hCLCA1, as analyzed by correlation protein expression graphics, detected a significant positive correlation between increasing levels of hCLCA1 and *Pneumocystis* burden suggesting induction by *Pneumocystis* (Spearman *r*=0.3479; *P*=0.0171) (Fig. 2). Common respiratory viruses were studied in *Pneumocystis*-positive and *Pneumocystis*-negative samples to assess their contribution to hCLCA1 expression levels. Respiratory Syncytial Virus was diagnosed in three and Adenovirus in one of the *Pneumocystis*-positive samples. No viruses were detected in the *Pneumocystis*-negative samples (Fig. 3). Protein expression levels of hCLCA1 were no different in virus-positive compared to virus-negative samples indicating that, in these samples, viruses do not explain the *Pneumocystis*-associated increased levels of this protein. Moreover, virus positive samples were grouped for this comparative analysis, and no significant difference in hCLCA1 expression was detected between virus-positive and virus-negative samples (*P*=0.7648) (Fig. 3).

## 4 Discussion

The increased hCLCA1 protein levels associated with *Pneumocystis* in infant lungs documented in this study provide additional evidence that *Pneumocystis* infection is associated with stimulation of the respiratory epithelium mucus-secretion-system in non-immunocompromised humans (Fig. 1). Furthermore, all infants in this study were mostly asymptomatic prior to death revealing the mild nature of this *Pneumocystis* infection, which is consistent with a state of symptomless “colonization”. An association between increased levels of the goblet cell mucin MUC5AC and *Pneumocystis* was reported in a previous study [2], and the association between hCLCA1 and MUC5AC, which is well-documented in animal models, has been recently reported in patients diagnosed with COPD [21].

Results also document that an increasing burden of *Pneumocystis* is correlated with increasing levels of hCLCA1 protein expression, suggesting a *Pneumocystis*-related, stimulatory effect on hCLCA1 induction (Fig. 2) [6,8]. Overexpression of hCLCA1 may explain the increase in mucus proteins, such as MUC5AC, associated with *Pneumocystis* through stimulation of the STAT6–hCLCA1–MUC5AC proposed pathway [7]. *Pneumocystis* is a slowly replicating fungus that may likely require days or even weeks to induce an effective T-cell lymphocytic response capable of clearing *Pneumocystis* from the lungs, therefore allowing time for prolonged stimulation of the respiratory epithelium and to induce the expression of *mClca3*/*Gob5* documented in rodents [11], and of hCLCA1 in this study in humans. Importantly, the induction of STAT6-dependent pathways by *Pneumocystis* recently documented in rodents can result in clinico-pathological consequences, including *Pneumocystis*-induced airway hyperresponsiveness (AHR) [12], therefore underscoring the need for further study of these mechanisms in human lungs.

Mucus-associated up-regulation by *Pneumocystis* may be theoretically relevant in different scenarios. For example, as a co-factor in increasing severity of respiratory illnesses during infancy, when narrow developing airways are present. *Pneumocystis* colonization is highly prevalent in infants, affecting over 90% of infants between 2 and 5 months of age, when respiratory morbidity typically increases [2]. Results may also strengthen the association between *Pneumocystis* and severity of COPD [3]; a disease that is strongly associated with increased mucus compromising narrow airways in immunocompetent adults [4,21,22]. Overexpression of *mClca3* induces mucous cell metaplasia, airway hyperreactivity (AHR) and increased airway resistance in immunocompetent rodents, and is also correlated with increased MUC5AC levels [6,7]. Recent studies using microarray technology on lung samples from patients diagnosed with COPD found *Pneumocystis*-related overexpression of proteins that are predominantly expressed on activated Th 1 T-lymphocytes showing the complexity of this *Pneumocystis* host interaction [23]. A more complete characterization of the immune response to *Pneumocystis* in these infants might lead to understand any potential role in disease. It is well known that respiratory viruses are associated with mucus hypersecretion, including increased expression of MUC5AC [4,6,7]. Therefore, we were expecting to detect an additive increase of hCLCA1 in samples where *Pneumocystis* and viruses were associated. Unfortunately, the limited number of virus-positive specimens detected using DNA/RNA amplification techniques in this study precluded us from identifying any relationship between common respiratory viruses and increased hCLCA1 or MUC5AC. The 20% pooled detection rate for viruses is similar to previous studies published by our group on autopsied infant lungs using viral cultures and immunofluorescence [24]. *Pneumocystis* is highly endemic in infants, while viruses follow epidemic patterns. Viruses are of low prevalence in these type of infant samples [25]. The viruses that were examined in these samples were the same as previously identified using other techniques [24]. Of note, hCLCA1 (and MUC5AC; data not shown) determination values in the virus-positive specimens of this study were close to average, and therefore indicate that viruses do not explain the increased expression of these proteins associated with *Pneumocystis* in infants (Fig. 3).

In summary, results show that the primary infection by *Pneumocystis* may play a role in up-regulating airway mucus-related responses in non-immunosuppressed infants through induction of an hCLCA1-related pathway. These type of responses may affect lung function, as shown in rodents, therefore suggesting that up-regulated airway epithelium innate responses may be clinically relevant to infants and the general population in different clinical scenarios where *Pneumocystis* is common. Further research to elucidate hCLCA1-related pathways associated with *Pneumocystis* infection in humans, and to assess the potential impact of *Pneumocystis* asymptomatic infection in respiratory disease of the immunocompetent host, is warranted.
